# Supplementary figures and images for: Enhanced protein synthesis is a defining requirement for neonatal B cell development
Source: Front Immunol. 2023 Apr 17;14:1130930. doi: 10.3389/fimmu.2023.1130930 (PMC10149930; doi:10.3389/fimmu.2023.1130930)

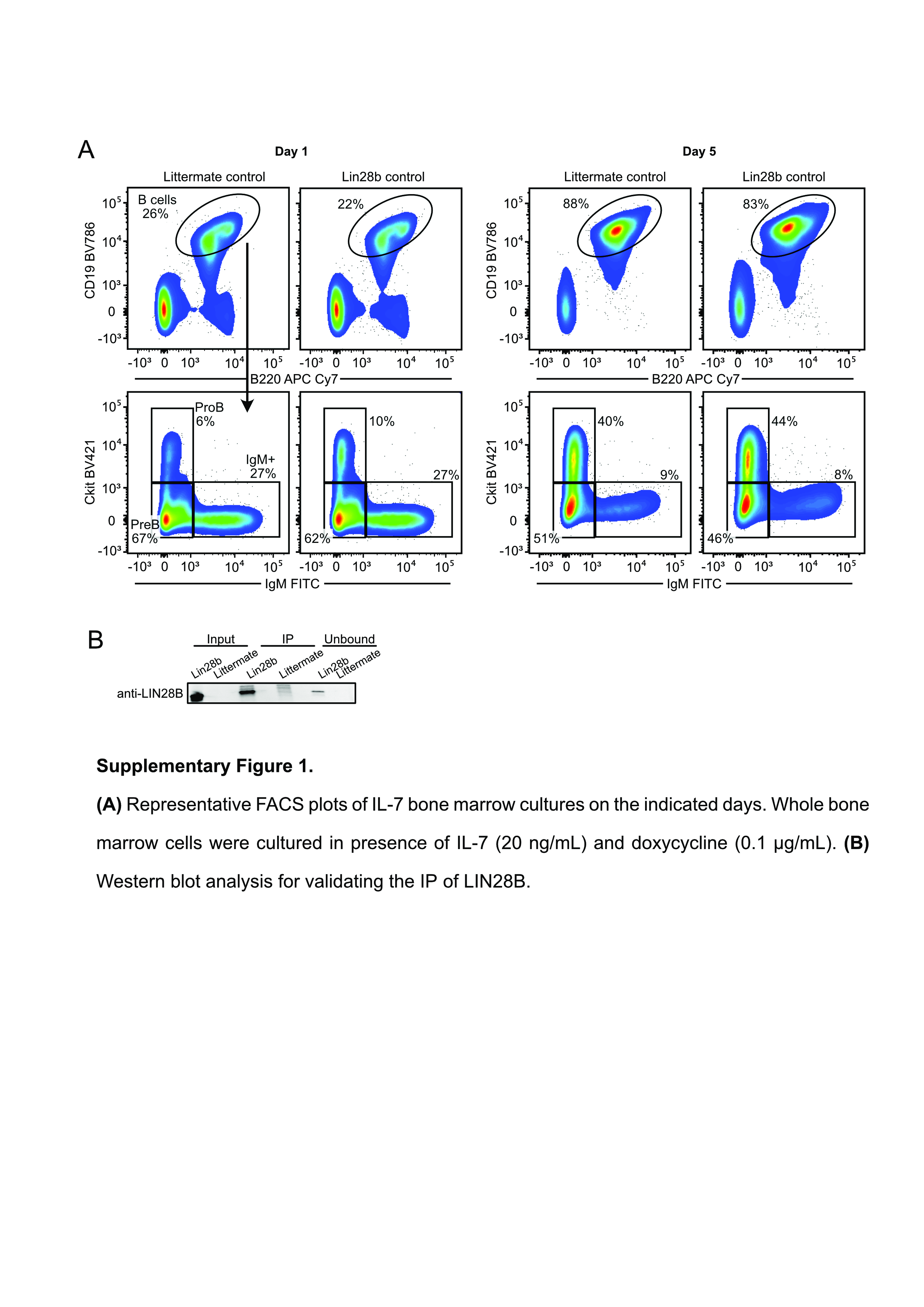

Supplement: Supplementary file 3 [file Image_1.tiff]

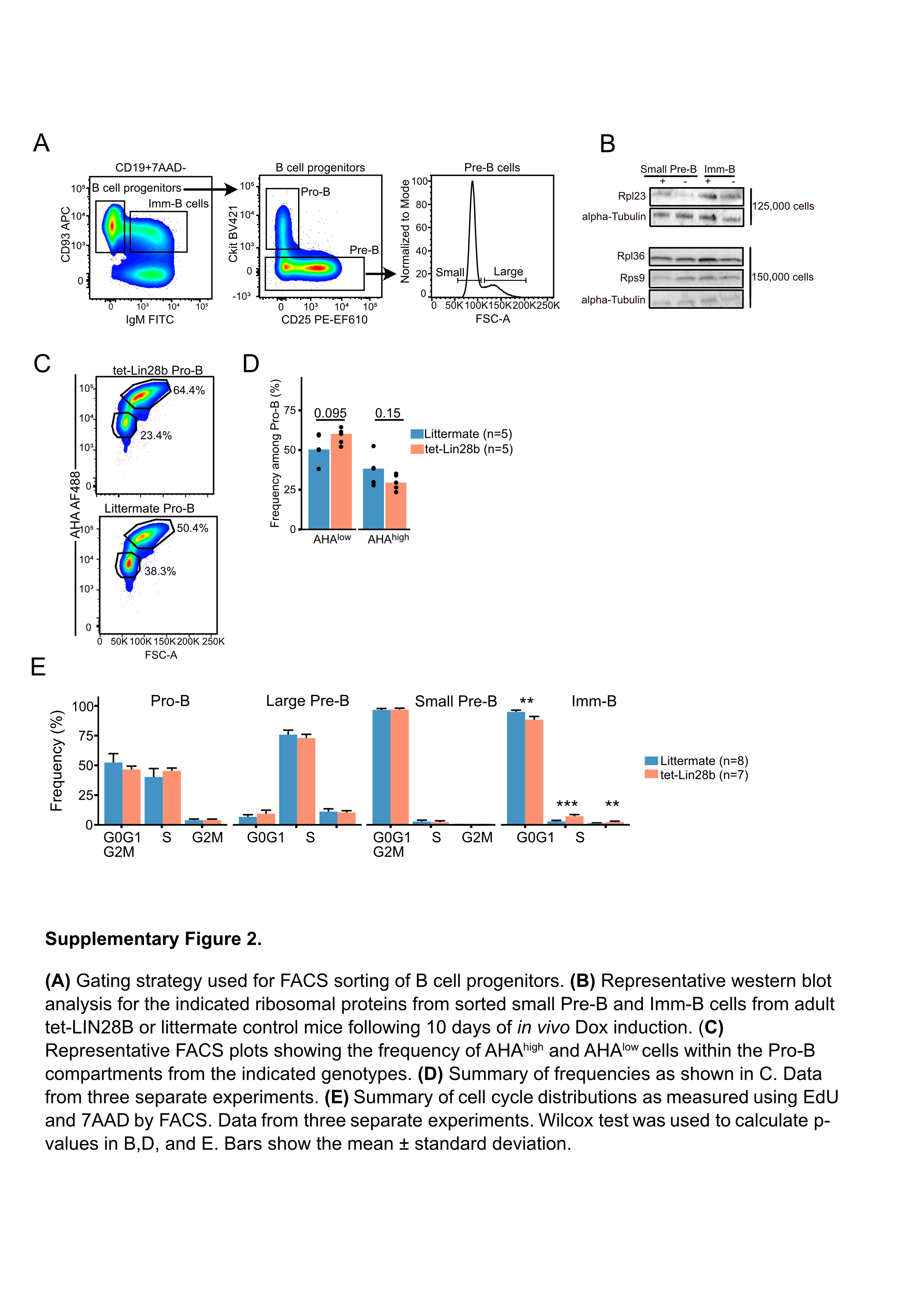

Supplement: Supplementary file 4 [file Image_2.tiff]

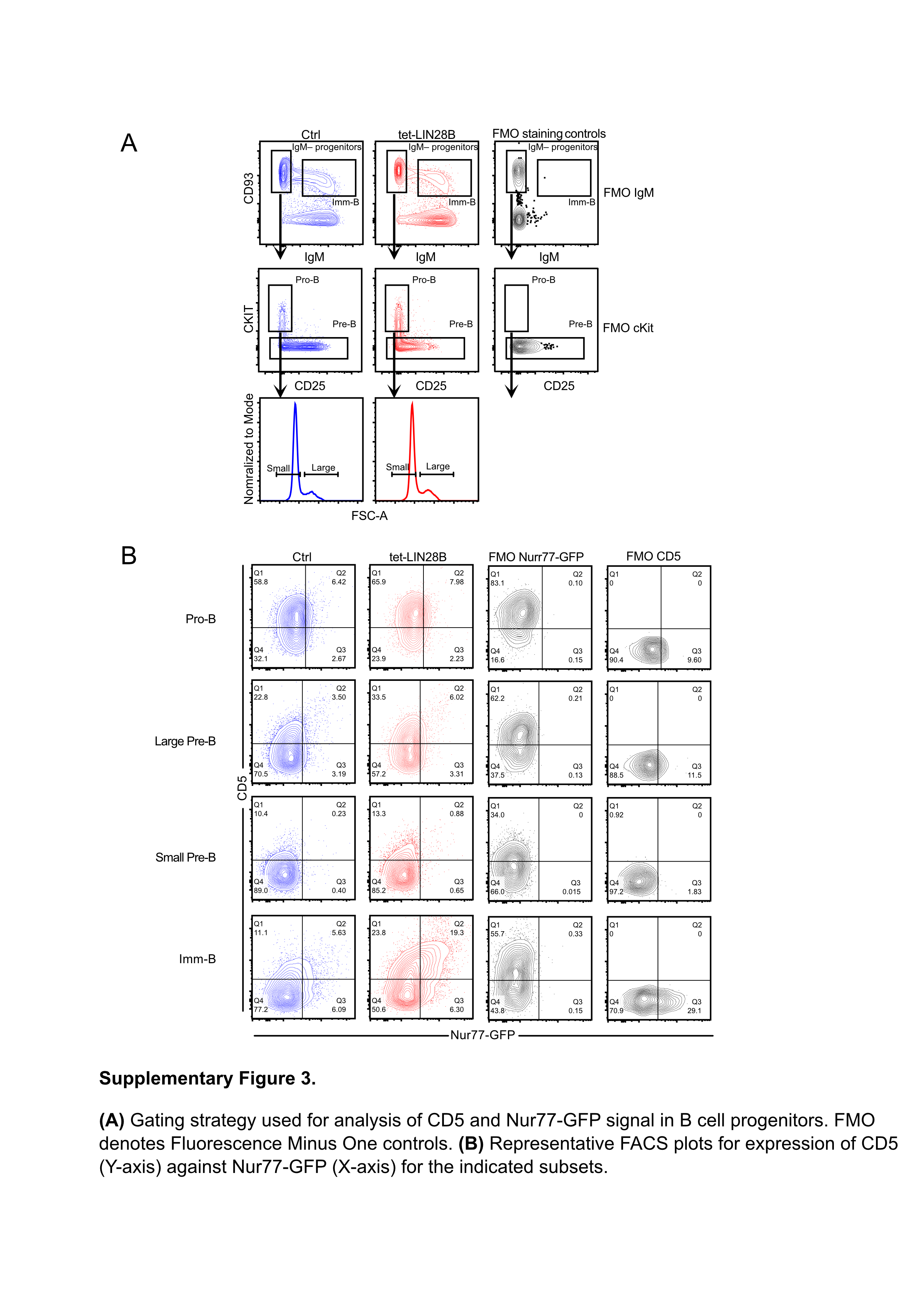

Supplement: Supplementary file 5 [file Image_3.tiff]
